# Detection of the Single-Session Complete Ablation Rate by Contrast-Enhanced Ultrasound during Ultrasound-Guided Laser Ablation for Benign Thyroid Nodules: A Prospective Study

**DOI:** 10.1155/2016/9565364

**Published:** 2016-11-23

**Authors:** Shuhua Ma, Ping Zhou, Xiaomin Wu, Shuangming Tian, Yongfeng Zhao

**Affiliations:** Department of Ultrasound, The Third Xiangya Hospital, Central South University, Changsha 410013, Hunan, China

## Abstract

This study aimed to investigate the single-session complete ablation rate of ultrasound-guided percutaneous laser ablation (LA) for benign thyroid nodules. LA was performed in 90 patients with 118 benign thyroid nodules. Contrast-enhanced ultrasound (CEUS) was used to evaluate complete nodule ablation one day after ablation. Thyroid nodule volumes, thyroid functions, clinical symptoms and complications were evaluated 1, 3, 6, 12, and 18 months after ablation. Results showed that all benign thyroid nodules successfully underwent LA. The single-session complete ablation rates for nodules with maximum diameters ≤2 cm, 2-3 cm and ≥3 cm were 93.4%, 70.3% and 61.1%, respectively. All nodule volumes significantly decreased than that one day after ablation (*P* < 0.05); at the final evaluation, the volume decreased from 6.16 ± 5.21 mL to 0.05 ± 0.01 mL. Thyroid functions did not show significant differences at one month after ablation compared with that before (*P* > 0.05). Three patients had obvious pain during ablation; one (1.1%) had recurrent laryngeal nerve injury, but the voice returned to normal within 6 months after treatment. Thus, ultrasound-guided LA can effectively inactivate benign thyroid nodules. LA is a potentially viable minimally invasive treatment that offers good cosmetic effects.

## 1. Introduction

Thyroid nodules are clinically common and occur frequently. High- frequency ultrasound examination can detect nodules of approximately 2 mm, and the detection rate can reach 20%–50% [1]. Most pathological results are benign, and only 5% of all nodules detected are malignant [2]. However, benign nodules will increase in volume by 15% in one year, and within 5 years, the volume increase may reach almost 89% [3]. Some nodules have significant compression symptoms because of their large volumes and some nodules may transform into malignancy. If they are not treated in time, the nodules may cause acute exacerbation of respiratory diseases, causing irreparable loss [4].

The conventional treatments for thyroid nodules are levothyroxine drugs and surgery to remove the thyroid gland. The clinical efficacy and safety of drug treatment is still controversial. Levothyroxine cannot inhibit the growth of all nodules, and it should not be used for elderly patients or postmenopausal women [5]. Although surgical treatment may be effective, there is a high incidence of complications, including airway obstruction, anesthetic accidents, scarring, recurrent laryngeal nerve injury and iatrogenic hypothyroidism [6]. In addition, drugs are needed to maintain the treatment for a period of time after surgery. Due to lack of gland function lifelong drug substitution is required after surgery, which seriously affects the life quality. Especially in the case of small-volume nodules, it is difficult for patients to decide whether to undergo surgery. Therefore, it is important to retain healthy glandular tissue that can continue to function while attempting to cure tumors. Minimally invasive treatments, such as ethanol injection, microwave ablation and radiofrequency ablation, have become popular. Preliminary results have been reported for their use in the treatment of thyroid nodules [7–9]. Ethanol injection can effectively reduce nodule volumes, but it also has inadequacies: for example, ethanol can easily penetrate toward the surrounding tissues and cause severe pain. After treatment, fibrosis clearly causes trouble for later surgeries. Most nodules require multiple repeated treatments, which limit the further clinical application of ethanol injection. The use of thermal ablation (radiofrequency and microwave) to treat thyroid nodules also has certain limitations. The thick electrode needle required for thermal ablation treatment carries risks because of the good blood supply of the thyroid, and there are vital tissues and organs, such as the trachea and the recurrent laryngeal nerve, surrounding the thyroid.

Laser ablation (LA) is a minimally invasive treatment. Currently, its successful use for treatment of liver cancer, lung cancer, pancreatic cancer and other tumors has been reported [10–12]. Recently, reports have supported the use of LA on thyroid lesions to achieve non-surgical targeted cytoreduction of tumor cells [13–18]. LA treatment of thyroid nodules has several advantages: it is minimally invasive, effective and does not leave scars on the neck. However, the use of LA to reduce thyroid nodules in volume had been largely reported, but very few studies examined the single-session complete ablation rate for thyroid nodules. Our study aimed to investigate the single-session complete ablation rate of ultrasound-guided percutaneous LA for benign thyroid nodules in 90 cases with 118 benign lesions.

## 2. Materials and Methods

### 2.1. Study Subjects

The present prospective study was conducted at the Third Xiangya Hospital of Central South University, Changsha, China. Ninety-five patients with a total of 125 lesions who were admitted to our hospital for LA treatment between September 2012 and April 2014 were included in this study. Inclusion criteria were as follows: (1) repeated benign cytological findings; (2) anxiety about malignant change, and not willing to follow up; (3) patients who had recurrence after surgery; (4) patients who had significant nodular oppression of surrounding organs, blood vessels and nerves or cosmetic problems and who refused surgeries. Exclusion criteria were as follows: (1) patients with coagulation disorders; (2) patients with malignant nodules; (3) patients with suspicious malignant nodules (marked hypoechoic areas, solid nodule, microcalcification, irregular margins, anteroposterior : transverse diameter ratio (A/T) > 1). Finally, 90 patients with 118 nodules met the inclusion criteria. The patient group included 34 males and 56 females with an average age of 41 years (range: 24–64). Seventy-six patients had single nodules, and 14 had multiple nodules. Sixty-nine nodules were located on the left lobe, 48 were located on the right lobe, and one was located in the isthmus; 75 were solid nodules, and 43 were mixed nodules. The maximum diameter of the nodules was between 1.36 and 4.72 cm, the volume of the nodules was between 0.72 and 34.9 mL. All nodules underwent preoperative puncture pathological examination and were found to be benign. Seventy-two were nodular goiters, and 46 were adenomatous goiter nodules. This study was approved by the Ethics Committee of the Third Xiangya Hospital, Central South University (20120901A), and all patients were informed and signed the consent forms.

## 3. Equipment

### 3.1. LA Equipment

A commercially available ultrasound system with an integrated laser source (EcholaserX4®, Elesta, Florence, Italy) was used for LA treatment and intraoperative monitoring throughout the process. The laser therapy equipment has a total of four leads and can perform up to four needle tract treatments simultaneously. The laser fiber has a length of 11 cm, a diameter of 300 *μ*m and a supporting guide needle of 21 G. Each laser fiber has its output power set as 3 W, and the output laser energy is determined by the size of the nodule.

### 3.2. Ultrasound System

A Siemens Acuson S2000 color Doppler ultrasonic diagnostic instrument (Siemens, Mountain View, CA, USA) with a 9L4 probe and a frequency of 4–9 MHz and a MyLab Twice color Doppler ultrasound diagnostic instrument (Esaote, Elesta, Florence, Italy) with an LA523 probe and a frequency of 4–13 MHz were used. Both of the machines were equipped with contrast-enhanced ultrasound (CEUS) imaging technology. The contrast agent used in the present study was SonoVue (Bracco, Milan, Italy), which contains sulfur hexafluoride microbubbles. Before and after ablation, conventional ultrasound, CEUS and regular postoperative follow-up reviews of the thyroid nodules were performed.

### 3.3. Pre-Ablation Assessment

All of the patients underwent conventional ultrasound, CEUS and lab examinations before treatment. Conventional ultrasonography showed the sites of the nodules, their quantities, sizes, volumes and contents and suspicious lymph node metastasis. The nodules were observed to determine their blood supply and their relationship with surrounding tissues. Three orthogonal diameters of the thyroid nodules (the largest diameter and the two perpendicular diameters) were measured. Nodules were divided into three groups based on their maximum diameter: ≤2 cm, 2-3 cm and ≥3 cm. The volume of the nodules was calculated using the following equation: *V* = *πabc*/6 (*V*: volume, *a*: the largest diameter, *b* and *c*: the other two perpendicular diameters). Nodules were divided into two categories based on their contents: solid-based nodules and mixed nodules. They were subjectively evaluated by sonographers. Solid-based nodules were nodules that were more than 80% solid with cystic component not exceeding 30% of the basal volume of the nodule; mixed nodules were with less than 80% solid. CEUS was used to examine the enhanced status and coagulation zone of the nodules [19].

### 3.4. Procedure

**Figure 1:**
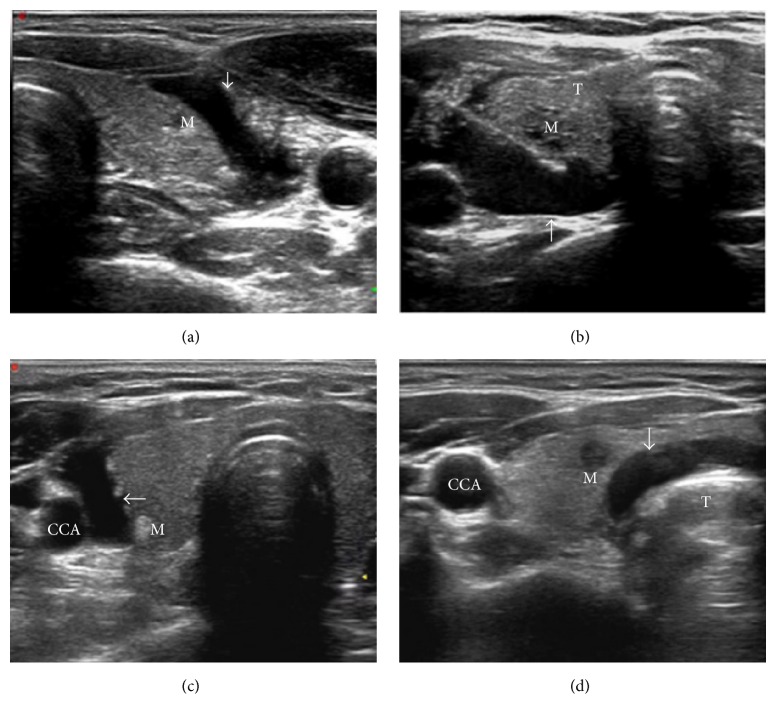
(a) A left lobe nodule near the front capsule; a “hydrodissection technique zone” (arrow) formed in front of the thyroid after liquid injection. (b) A right lobe nodule near the thyroid back capsule; a “hydrodissection technique zone” (arrow) formed at the back of the thyroid after liquid injection. (c) A right lobe nodule nears the carotid; a “hydrodissection technique zone” (arrow) formed outside the thyroid after liquid injection. (d) A right lobe nodule close to the trachea; a “hydrodissection technique zone” (arrow) formed inside the thyroid after liquid injection.

The patient was placed in the supine position with the hyperextended neck. Local anesthesia consists of 2% subcutaneous Lidocaine, followed by subcapsular infiltration of Lidocaine (2–5 mL) under US guidance. A 21-gauge Chiba needle was placed into the nodules along the selected route. Then optical fiber was inserted. When the fiber was parallel to the sheath of the Chiba needle, the sheath was retreated with 5 mm of the bare fiber being in direct contact with thyroid tissue. For nodules that were close to the carotid artery, trachea, esophagus and the path of the recurrent laryngeal nerve, a mixture of 0.9% lidocaine and physiological saline (10–30 mL) was infused into the surrounding thyroid capsule to achieve a “liquid isolating region” by hydrodissection technique with at least a 5 mm width between the nodules and the abovementioned organs (Figure 1).

**Figure 2:**
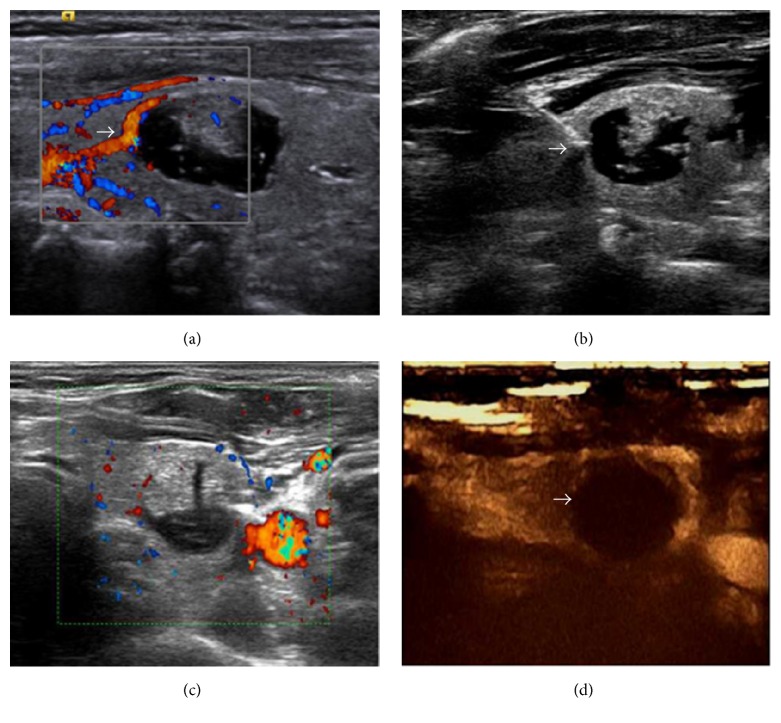
(a) Thick feeding vessels can be seen surrounding left lobe nodules of thyroid before LA. (b) A laser is used to occlude feeding vessels of the nodules before LA. (c) Peripheral and internal nodule blood flow disappeared after occlusion of feeding vessels. (d) Immediate CEUS showed no enhancement inside the nodules after occlusion of feeding vessels.

Treatment strategies were determined based on nodular size, cystic or solid content and blood flow richness. For nodules with volume ≤15 mL, single fiber was used; for solid nodules with volume >15 mL, multiple fiber was used; for mixed cystic and solid nodules with volume >15 mL, we first drained the cyst fluid and then performed single- fiber or multiple- fiber ablation based on the nodular volume after draining. If the nodular blood flow was very rich, the “heat sink effect” of the blood flow could affect the ablation effect. Therefore, we first occluded the surrounding major feeding vessels (Figure 2), which can both avoid the “heat sink effect” and reduce the potential risks caused by bleeding during treatment. Then single- or multiple- fiber ablation was performed according to nodular size. During the ablation, the initial energy is delivered at 1,200 Joules per fiber with an output power of 3 W and the energy differed according to the nodule size. After laser activation, ultrasonography showed highly echogenic area, and vaporization was observed. As soon as the energy was deployed into the nodule, the highly echogenic area gradually expanded to the surrounding tissue and covered the entire nodule. Vital signs were monitored throughout the operation process. We asked the patients about pain conditions intermittently throughout the operation and determined the condition of the patients' vocal cords based on their voice when they answered. The therapy was not stopped until the highly echogenic area covered the whole nodule.

### 3.5. Post-Ablation Care and Efficacy Evaluation

After treatment, the patient was observed for 30 minutes. The patient discharged after one day of proper supportive treatment. CEUS evaluation was used one day after ablation to calculate the nodule ablation ratios. The post-ablation area was measured and compared with the nodular area before LA treatment to evaluate whether the original nodule was completely covered. If there was any residue in the boundary, supplementary ablation was performed on a selected later day.

### 3.6. Follow-Up Evaluation

Regular follow-up was performed 1, 3, 6, 12 and 18 months after ablation. The follow-up included evaluation with conventional ultrasound, lab examinations and clinical examinations. Conventional ultrasound was used to evaluate the ablated nodular volume change, echogenicity, and vascularity. The volume reduction rate was calculated using the following equation: Volume reduction ratio (%) = [(initial volume − final volume) × 100]/initial volume. The immediate post ablation size was used as the initial volume to determine size reduction. The lab examinations included thyroid function and the determination of autoantibodies related to thyroid tissues (FT3, FT4, TSH).

### 3.7. Statistical Analysis

Statistical analysis was performed using SPSS 18.0 statistical software. Quantitative data for the maximum diameter of the ablation area, the volume and the volume reduction ratio were analyzed using a paired *t*-test. The ablation rate was analyzed using Pearson's *χ* ^2^, and the significance level (*α*) was 0.05.

## 4. Results

### 4.1. Post-Ablation Ultrasound Evaluation

**Figure 3:**
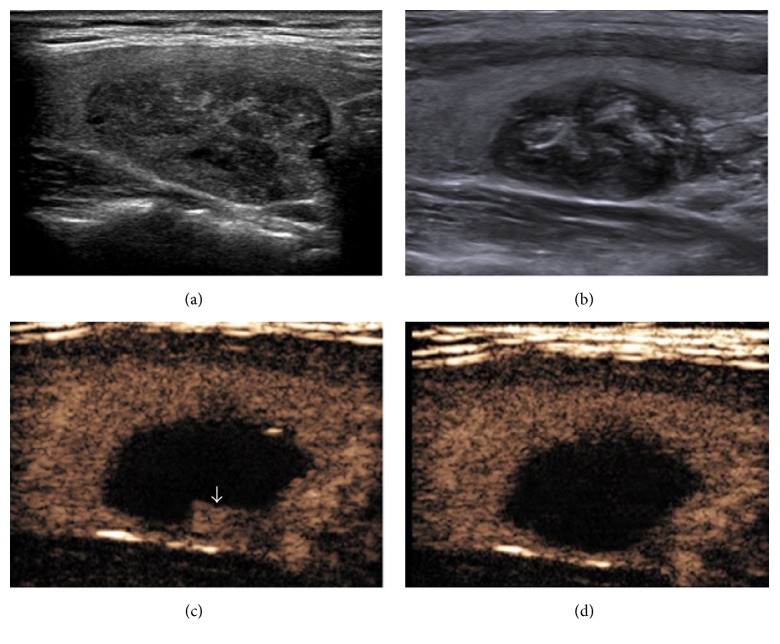
A 36-year-old female had a solid nodule in the left lobe of the thyroid. (a) Ultrasound examination showed a 25 × 17 mm hypoechoic nodule before LA. (b) Ultrasound examination showed a heterogeneous ablation lesion one day after LA. (c) CEUS showed residue at the edge (arrow) after the first ablation. (d) CEUS showed no enhancement in the residual part after the second ablation.

Two-dimensional ultrasonography one day after ablation showed that the nodule had a hyperechoic scar without posterior acoustic shadowing in the center, hypoecho in the peripheral tissues and clear boundaries to the normal tissues. Color Doppler showed rich colorful blood flow signals inside and at the edge of nodules before treatment. Blood flow inside the nodules disappeared after the treatment, and blood flow around nodules decreased or disappeared. After single LA session, CEUS showed that the complete ablation rates of the three groups of nodules with different sizes differed (Table 1). In the nodule ≤2 cm group, except for 3 nodules that had no complete ablation, all other nodules had no enhancement or no contrast agent filling. The coagulation zone was bigger than the original nodule, with clear boundaries, and the single-session complete ablation rate was 93.4%. In the 2-3 cm group, the single-session complete ablation rate was 70.3%; and in the ≥3 cm group, the single-session complete ablation rate was 61.1%. The ≤2 cm and 2-3 cm groups had no significant difference in ablation effects (*P* > 0.05), and 2-3 cm and ≥3 cm groups had no significant difference in ablation effects (*P* > 0.05). However, the ≤2 cm and >3 cm groups showed a significant difference in ablation effects (*P* < 0.05). CEUS showed that incompletely ablated nodules had a small amount of irregular residual contrast agent perfusion tissues at the edge, and the residual part was completely ablated after another ablation (Figure 3).

### 4.2. Side Effects and Complications of the Ablation

The side effects of LA treatment are limited to local pain, local subcutaneous soft tissue edema and fever. Patients had intraoperative mild distending pain in the neck. The pain was tolerable, and the whole ablation operation was finished with no special treatment. Two patients had intraoperative intense pain, and the pain decreased after ablation was stopped; after a short break, the ablation was continued and completed without the use of painkillers. Immediate postoperative observation showed a small needlepoint on one patient's neck skin, slight swelling of the surrounding subcutaneous tissues, without occurrence of neck structure movement disorders or muscle and skin burns. One patient mentioned pain in the ablated area three hours after operation, with pain radiating to the head and face. No painkillers were used, and the untreated pain disappeared spontaneously 7 days after operation. Two patients with large nodular volumes had a mild fever that disappeared naturally after three to four days. No trachea, esophagus or blood vessel damage or serious complications, such as hematoma and local infections, were observed in any of the patients. One patient reported coughing during drinking and a hoarse voice after ablation. After physical and drug treatment, coughing during drinking recovered after a week, and the voice was back to normal after six months. None of the patients experienced complications such as iatrogenichypothyroidism or hypoparathyroidism.

### 4.3. Post-Ablation Follow-Up

**Figure 4:**
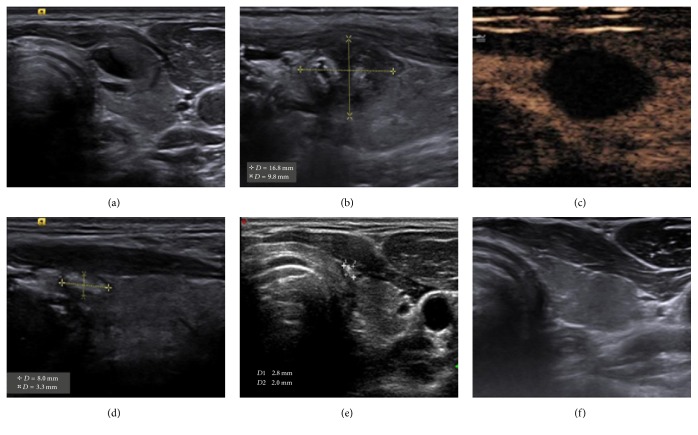
A 33-year-old Male had a mixed nodule in the left lobe of the thyroid. (a) Ultrasound examination showed a 13 × 8 mm mixed nodule protruding from the front capsule before LA. (b) Ultrasound examination showed a 16.8 × 9.8 mm ablation lesion one day after LA. (c) CEUS showed no enhancement of the ablation lesion one day after LA. (d) Six months after LA, Ablation lesion had an obvious volume reduction, and no prominent capsule was observed. (e) Nine months after LA, ablation lesion had an obvious volume reduction compared with the six-month results. (f) 12- month after LA, the ablation lesion had disappeared.

None of the nodules had increased volume or needed surgical treatment at the last follow-up. The ablated nodular maximum diameter reduced from the immediate post ablation size 1.76–4.92 cm to 0.00–0.78 cm at the end of follow-up, and the volume reduced from the immediate post ablation size 7.41 ± 5.19 mL to 0.05 ± 0.01 mL at the 18-month follow-up. The average volume reduction ratios during follow-up at 1, 3, 6, 12 and 18 months later were 42.3%, 53.5%, 77.1%, 96.5% and 98.8%, respectively. The ablated nodular maximum diameter, volume and volume reduction ratio showed significant differences (*P* < 0.05) (Table 2; Figure 4). The thyroid function examination showed that one day after ablation, serum TSH significantly decreased, but FT4 significantly increased. During the follow-up at one month after ablation, FT4 and TSH had both recovered to normal level and were stable (*P* > 0.05).

## 5. Discussion

Surgical resection is the most commonly used clinical treatment for thyroid nodules. However, surgical resection has inadequacies, such as considerable trauma and aesthetic effects. Therefore, the use of surgical resection to treat benign thyroid nodules has been controversial. Active follow-up observation should mainly be used for thyroid nodules that do not cause clinical symptoms, especially small nodules that do not require surgical resection. However, this approach does not address patients' anxieties about the nodules. Moreover, benign nodules have a 5% possibility of malignancy, which often induces a sense of passive waiting and insecurity in patients. Therefore, finding a minimally invasive method to effectively treat thyroid nodules is an important issue in the current clinical field.

Ultrasound-guided percutaneous LA has been successfully used as a minimally invasive technology for the treatment of thyroid nodules. Pacella et al. [20] performed experimental LA treatment for 18 thyroid resection specimens and 2 high-functioning thyroid tumors, and proved that using LA to treat thyroid nodules is feasible. Later, Døssing et al. [21–23] carried out LA treatment 3 times for 16, 30 and 78 cases of benign cold thyroid nodules. The results showed that nodular volume reduction ratios six months after treatment were 46%, 44% and 51%, respectively, suggesting that laser treatment has good effects for tumor reduction. Valcavi et al. [24] reported 122 benign solid thyroid cold nodule patients and maintained a three-year follow-up. The average volume reduction ratio was 47.8%, and 73% of the patients had significantly improved clinical symptoms. These results indicate that LA can effectively treat thyroid nodules. However, the above studies mainly examined the use of laser treatment to reduce thyroid nodules. No reports investigate the single-session complete ablation rate of ultrasound-guided percutaneous laser ablation (LA) for benign thyroid nodules.

In this study, we divided the nodules into three groups based on size. The results showed that LA had the best single-session ablation effect for ≤2 cm small nodules, with a complete ablation rate of 93.4%; and ≥3 cm nodules had the lowest ablation rate of 61.1%. The nodules that were ≥3 cm had a significantly lower complete ablation rate than the other two nodule groups (*P* < 0.05). The major reason for the incomplete ablation of the nodule in the ≤2 cm group is that the nodules were close to the recurrent laryngeal nerve, and for safety reasons, the edge was unablated. In the 2-3 cm group, ablation was incomplete. Two of these were close to the recurrent laryngeal nerves; the others had a large size (close to 3 cm), and small residues were left at the nodule edge. The nodules >3 cm had larger volumes and usually could not be completely ablated in one attempt. For large nodules >2 cm, we still need to actively explore strategies for expanding ablation. To improve the complete ablation rate for >2 cm nodules, our group actively explored strategies of expanding the coagulation zone: for mainly cystic nodules, the cyst fluid was first aspirated, and then LA was performed; For nodules with a rich blood flow, the feeding vessels were first occluded, and then ablation of the tumors was performed. Both methods had very good therapeutic effects. In addition, 20 months of follow-up for the ablated nodules was performed. Results showed that nodular volumes were gradually reduced; the nodular volume reduction ratios one, three, six and 12 months after ablation were higher than the reduction ratios reported by Døssing et al. [21–23]. The reason for this difference could be that, on the one hand, complete ablation was used; on the other hand, most of the nodules we chose had a smaller volume, and the necrotic tissues could be easily absorbed after ablation.

To ensure complete ablation, it is also important to estimate the coagulation zone during the ablation. Our preliminary determination was based primarily on highly echogenic area coverage, which has the advantages of convenience and real-time information. However, this determination method can also cause errors. Because the highly echogenic area is not always entirely consistent with the actual coagulation zone, we recommend to estimate the coagulation zone while considering the fiber experimental data. For example, with the output power at 3 W and the energy at 1200 J, we generally considered the largest maximum long diameter of 1.8 cm, the traverse diameter of a 1.0 cm ellipsoidal ablation lesion. Of course, we also evaluated the coagulation zone by using intraoperative CEUS. However, intraoperative CEUS is often affected by the gases produced by ablation. In many cases, ideal results can only be achieved after the gas dissipates. Postoperative CEUS is the main evaluation method for LA treatment. All of the patients in this group underwent CEUS one day after the operation to verify whether the nodules were completely inactivated. If CEUS showed nodules with a small amount of residual tissues at the edge, then the patient required further ablation treatment until the remaining lesions disappeared completely. Our group did not have completely ablated nodules after one LA treatment. After the second ablation, all of the nodules were completely inactivated.

LA treatment of thyroid nodules has good safety and good efficacy [25, 26]. Our group totally ablated 118 lesions. Only three patients had obvious pain, two patients had fever, and one patient had recurrent laryngeal nerve injury. The reason for recurrent laryngeal nerve injury could be that the nodule was too close to the dorsal recurrent laryngeal nerve. During ablation, the excessive pursuit of one-time complete nodule ablation can cause nerve injury. For nodules located in high-risk areas, attention should be given to the following aspects that can significantly reduce the complication rate: (1) Ensure that vital organs are located a safe distance from the nodule (forward > 10 mm, side > 5 mm). (2) Inject lidocaine saline solution between the thyroid gland and the vital organs, generating a liquid isolating region by hydrodissection technique. (3) Insert a temperature measuring needle between the thyroid gland and the vital organs, and perform real-time temperature measurement. (4) If the nodules are too close to vital organs, do not over pursue complete ablation of the entire nodule. Appropriately reduce the energy output close to vital organs. (5) If both thyroid side lobes have nodules, carry out LA on the nodule on one side at a session to avoid nerve injury of both sides that cannot be compensated.

There are some limitations in this study. First, only benign thyroid nodules <5 cm were studied, many benign thyroid nodules >5 cm were not included in this study. Second, follow-up period was short. Third, the number of small nodules (less than 2 cm) is 39% (46/118) of all cases, which may be an important cause of excellent volume reduction of this study compared with previous studies. Hence, further studies with a larger sample size and long-term follow-up will be necessary.

## 6. Conclusions

In summary, LA has the advantages of being safe, effective and minimally invasive, can effectively inactivate benign thyroid nodules. The successful application of new LA technology for thyroid nodule treatment will greatly enrich thyroid nodule treatment methods. Laser treatment can be used as a supplement for surgical treatment.

## Figures and Tables

**Table 1:** CEUS evaluation of the nodular single-session complete ablation rate after LA.

Nodule size (cm)	Ablated nodule (*n*)	Completely ablated nodule (*n*)	Ablation rate (%)
≤2	46	43	93.4
2-3	54	38	70.3
≥3	18	11	61.1

**Table 2:** The changes in volume after LA and at each follow-up.

	Nodule numbers (*n*)	Maximum diameter (cm)	Volume (mL)	Volume reduction ratio (%)
Before ablation	118	1.36–4.72 (2.51 ± 0.68)	0.72–34.9 (6.16 ± 5.21)	—
One day later	118	1.76–4.92 (2.45 ± 0.87)	1.78–38.4 (7.41 ± 5.19)	—
One month later	104	1.48–3.95 (2.43 ± 0.57)	0.05–20.6 (4.31 ± 3.01)	42.3
Three months later	96	1.21–3.72 (2.21 ± 0.67)	0.74–17.5 (3.61 ± 2.59)	53.5
Six months later	75	1.06–3.12 (1.74 ± 0.39)	0.41–8.41 (1.61 ± 1.01)	77.1
Twelve months later	52	0.00–1.28 (0.68 ± 0.17)	0.00 ± 0.29 (0.05 ± 0.04)	96.5
Eighteen months later	48	0.00–0.78 (0.15 ± 0.06)	0.00–0.12 (0.05 ± 0.01)	98.8
